# Psychological Effects of a 1-Month Meditation Retreat on Experienced Meditators: The Role of Non-attachment

**DOI:** 10.3389/fpsyg.2016.01935

**Published:** 2016-12-12

**Authors:** Jesus Montero-Marin, Marta Puebla-Guedea, Paola Herrera-Mercadal, Ausias Cebolla, Joaquim Soler, Marcelo Demarzo, Carmelo Vazquez, Fernando Rodríguez-Bornaetxea, Javier García-Campayo

**Affiliations:** ^1^Faculty of Health and Sport Sciences, Primary Care Prevention and Health Promotion Research Network, Zaragoza, Centro de Investigación Biomédica en Red de Salud Mental Zaragoza, Spain; ^2^Centro de Investigación Biomédica en Red de Salud Mental, Primary Care Prevention and Health Promotion Research Network, Instituto Aragonés de Ciencias de la Salud Zaragoza, Spain; ^3^Department of Personality, Evaluation and Psychological Treatment, Universitat de València Valencia, Spain; ^4^CIBERObn Ciber Physiopathology of Obesity and Nutrition Santiago de Compostela, Spain; ^5^Servei de Psiquiatria, Hospital de la Santa Creu i Sant Pau (Barcelona), Departamento de Psicologia Clínica i de la Salut, Universitat Autònoma de Barcelona, Barcelona, Spain Centro de Investigación Biomédica en Red de Salud Mental, CIBERSAM Madrid, Spain; ^6^Mente Aberta – Brazilian Center for Mindfulness and Health Promotion, Department of Preventive Medicine, Universidade Federal de Sao Paulo Sao Paulo, Brazil; ^7^Hospital Israelita Albert Einstein Sao Paulo, Brazil; ^8^Professor of Psychopathology, Universidad Complutense de Madrid, Red PROMOSAM Valencia, Spain; ^9^Psychologist and Vipassana Master, President of Baraka Institute San Sebastián, Spain; ^10^Miguel Servet Hospital and University of Zaragoza, Primary Care Prevention and Health Promotion Research Network, Instituto de Investigación Sanitaria Aragón (IIS Aragon), Centro de Investigación Biomédica en Red de Salud Mental, Zaragoza, Spain

**Keywords:** meditation, Vipassana, retreat, wellbeing, positive psychology, personality

## Abstract

**Background:** There are few studies devoted to assessing the impact of meditation-intensive retreats on the well-being, positive psychology, and personality of experienced meditators. We aimed to assess whether a 1-month Vipassana retreat: (a) would increase mindfulness and well-being; (b) would increase prosocial personality traits; and (c) whether psychological changes would be mediated and/or moderated by non-attachment.

**Method:** A controlled, non-randomized, pre-post-intervention trial was used. The intervention group was a convenience sample (*n* = 19) of experienced meditators who participated in a 1-month Vipassana meditation retreat. The control group (*n* = 19) comprised matched experienced meditators who did not take part in the retreat. During the retreat, the mean duration of daily practice was 8–9 h, the diet was vegetarian and silence was compulsory. The Experiences Questionnaire (EQ), Non-attachment Scale (NAS), Positive and Negative Affect Schedule (PANAS), Satisfaction With Life Scale (SWLS), Temperament Character Inventory Revised (TCI-R-67), Five Facets Mindfulness Questionnaire (FFMQ), Self-Other Four Immeasurables (SOFI) and the MINDSENS Composite Index were administered. ANCOVAs and linear regression models were used to assess pre-post changes and mediation/moderation effects.

**Results:** Compared to controls, retreatants showed increases in non-attachment, observing, MINDSENS, positive-affect, balance-affect, and cooperativeness; and decreases in describing, negative-others, reward-dependence and self-directedness. Non-attachment had a mediating role in decentring, acting aware, non-reactivity, negative-affect, balance-affect and self-directedness; and a moderating role in describing and positive others, with both mediating and moderating effects on satisfaction with life.

**Conclusions:** A 1-month Vipassana meditation retreat seems to yield improvements in mindfulness, well-being, and personality, even in experienced meditators. Non-attachment might facilitate psychological improvements of meditation, making it possible to overcome possible ceiling effects ascribed to non-intensive practices.

## Introduction

Mindfulness is an umbrella term that can be understood to refer to the self-regulation of attention to one's experiences in the present moment with curiosity, openness, and acceptance (Bishop, 2004). Mindfulness practices can designate both contemporary approaches (such as mindfulness-based interventions) and traditional practices (such as Vipassana meditation), and they may modify many psychological and biological variables in clinical and non-clinical populations. These effects include reductions in stress (Demarzo, 2014; Sharma and Rush, 2014), cognitive rigidity (Greenberg et al., 2012), blood pressure and interleukin-6 (Tomfohr, 2015), and improvements in motor performance (Naranjo and Schmidt, 2012), quality of life (Demarzo, 2014), and emotional outcomes (Arch and Landy, 2015). Data on the effects of mindfulness on personality traits are still scarce, but it appears that it may shape individuals' personalities toward healthier profiles (Crescentini and Capurso, 2015). For instance, it has been described that impulsivity and self-maturity could be modified by mindfulness interventions (Peters, 2011; Van Dam, 2011; Soler, 2012, 2016; Crescentini and Capurso, 2015), and strong and negative associations between mindfulness and neuroticism have been reported (Giluk, 2009). On the contrary, mindfulness correlated strongly and positively with conscientiousness. Other positive—but weaker—correlations have been found between mindfulness and agreeableness, openness to experience and extraversion (Giluk, 2009).

There are few controlled studies on the effect of intensive mindfulness retreats on psychological variables in healthy long-term meditators. The main reason for this lack of research seems to be that these retreats do not typically use mindfulness-based interventions aimed at improving well-being or specific features in healthy people (such as empathy for clinicians, or burnout prevention for teachers). Instead, these types of retreats are typically delivered for highly motivated persons with an intensive meditation and/or religious practice (yogis, Buddhists, or contemplative practitioners from different schools). Therefore, the assessment of the psychological effects of these interventions is, in most cases, a secondary consideration. In addition, the heterogeneity of these studies is substantial, with great variations in terms of duration (from 1 week to several months), type of meditation (Vipassana, Yoga, Zen), and outcomes (Kozasa, 2008; Braboszcz, 2013; Jacobs, 2013; Elliott et al., 2014).

Vipassana describes a kind of meditative practice in which mindfulness of breathing and of thoughts are being used to gain insight into the true nature of reality (King, 1992). It is part of the deconstructive family of meditations, which has as an objective the reversal of maladaptive cognitive patterns by exploring the dynamics of perception, emotion, and cognition, generating insights into one's internal models of the self, the others and the world (Dahl et al., 2015). Usually, practitioners begin focusing attention on a chosen object or event (e.g., breathing). To maintain this focus, the practitioner has to monitor the concentration on the chosen event in order to avoid mind wandering (Tops, 2014). Focused attention is a first step that enables the practitioner to move forward to other methods and techniques, such as open monitoring, the main practice of Vipassana. During open monitoring, the focus of the meditation becomes the monitoring of awareness itself (Vago and Silbersweig, 2012). It has been observed that during a 1-month Vipassana meditation retreat, trained participants—compared to matched controls—exhibited improvements in response inhibition accuracy and reductions in reaction time variability. The trained group also reported increases in concentration during task performance, but not in effort or motivation (Zanesco, 2013). Another study involving a 10 day Vipassana retreat showed improvements in both well-being and heart rate variability (Krygier, 2013).

A number of studies on the psychological effects of different kinds of retreats have evaluated attentional and cognitive domains. A 1 week intensive retreat, based on mixing mindfulness and other therapeutic interventions, showed improvements in task-based indices of executive attention but not in reflexive or volitional orienting (Elliott et al., 2014). Another study, based on a 3-month Isha yoga meditation retreat, resulted in small but significant changes in some behavioral psychophysical tests (Braboszcz, 2013). On the other hand, other studies have evaluated a number of physiological variables. For example, a 3-month Samatha meditation retreat demonstrated an increase in self-reported mindfulness and a decrease in cortisol (Jacobs, 2013), and a 3-month concentrative meditation retreat resulted in an increase in cell telomerase activity and psychological mediators (Jacobs, 2011). Finally, one study involving a 1-week retreat with Metta training assessed different psychological variables, such us compassion and resilience, finding significant improvements at a 4-month follow up (Pidgeon et al., 2014).

However, few studies have been devoted to assessing the impact of mindfulness-intensive retreats on variables related to positive psychology. The small number of papers on this subject have confirmed an increase in positive affect (Shapiro et al., 2007) and in the enjoyment of positive sensory stimuli, such as pleasant film clips (Erisman and Roemer, 2010). However, other studies have suggested that usual mindfulness interventions may decrease individuals' emotional response to positive sensory stimuli (Ortner et al., 2007; Brown et al., 2013). The reason for this apparent contradiction is unclear, although a possible explanation may be that the effect of an intensive mindfulness retreat might be quite different, or at least more intense, from usual mindfulness practices. According to descriptions given by traditional meditation masters (Karmapa, 1981), the highest meditation states cannot be achieved only by daily practice in the midst of our busy world, but by intensive meditation retreats in an isolated environment.

Non-attachment is said to be one of the most salient correlates of mindfulness practice (Tran, 2014). It refers to a subjective quality characterized by an absence of fixation on ideas, images or sensory objects, as well as an absence of internal pressure to obtain, hold, avoid or change circumstances or experiences (Sahdra et al., 2010). According to Buddhist philosophy, a change in the perspective on the self is key to explain the effects of meditation (Gunaratana, 2009). In this sense, non-attachment has been proposed as one of the main adaptive action mechanisms in the improvement of well-being linked to mindfulness interventions (Hölzel, 2011; Tanay et al., 2012), and it has been used as a measure of the detached perspective of the self (Hölzel, 2011). It is related to an individual's amount of meditation practice (Tanay et al., 2012; Desbordes, 2015), and is able to differentiate between clinical populations, healthy people, and meditators (Sahdra et al., 2010; Sahdra, 2015).

In this context, despite the relative lack of previous controlled studies on the effect of long-term meditation retreats on positive psychological variables, we aimed to explore whether a 1-month Vipassana retreat: (1) would increase mindfulness and psychological well-being; (2) would increase prosocial personality traits; and (3) psychological changes produced by meditation would be mediated and/or moderated by non-attachment. In other words, whether higher basal levels of non-attachment (moderating effect) and greater improvements in this variable by means of meditative practice (mediating effect) would lead meditators to experience more benefits in psychological terms.

## Methods

### Design

A controlled, non-randomized, pre-post-intervention trial was conducted.

### Participants

The intervention group was a convenience sample (*n* = 19) consisting of individuals who had participated in a 1-month Vipassana meditation retreat that was led by Master Dhiravamsa and held at the Bujedo Monastery (province of Burgos, Spain), between August and September 2014. All retreatants were invited to participate in the study. The inclusion criteria were: (a) ability to understand and write Spanish, (b) willingness to participate in the study and signing an informed consent. The exclusion criteria were: (a) limited fluency in Spanish, and (b) suffering from a psychiatric or medical disorder that would impede the completion of the protocol. From the total of 23 participants in the retreat, 4 (17.39%) willingly decided not to participate in the study. No retreatants were ruled out for limited knowledge of Spanish or for suffering from a psychiatric or medical disorder.

The control group (*n* = 19) was recruited from the meditators who had attended any of the mindfulness courses given by our group during the last 3 years, linked to a master's degree course. This group was matched to the intervention group with regard to gender, age (±5 years), ethnic group, educational level, and type of meditative practice. The same inclusion/exclusion criteria described for the retreatants were used for this group. From the total of 21 who were approached to participate in the study, only 2 (9.52%) individuals decided not to do so, and no individual was ruled out for meeting the exclusion criteria.

### Intervention

The meditation practiced during the retreat was Vipassana, which mainly includes open monitoring meditations and focused attention (Lippelt et al., 2014). The mean duration of daily practice was 8–9 h, with 1–2 h of teaching and questions/answers. Meditative practice was mainly unguided. Practice was as a group during the first and fourth weeks, whereas practice was individual, done in each retreatant's own room, during the second and third weeks. The diet was vegetarian, and silence was compulsory. No contact (including by mobile or email) with the outer world was allowed.

The control group was asked not to participate in any retreat (even as short as a 1-day retreat) during the 1-month study period. Their usual daily meditation practice was maintained (40–50 min/session).

### Assessments

- In addition to general *socio-demographics* (age, sex, marital status, education, body mass index, hand dominance), a questionnaire on meditation practice was administered, asking for the main type of meditation practice, as well as years and duration of daily practice (an overall measure of estimated hours of practice was derived from data provided for the previous two variables).
- *Non-attachment Scale* (NAS; Sahdra et al., 2010): This is a 30-item questionnaire that is rated using a Likert scale between 1 (completely disagree) and 6 (completely agree). It measures the Buddhist notion of “non-attachment,” or release from mental fixations based on an insight into the constructed and impermanent nature of mental representations. Evidence consistent with Buddhist theory has demonstrated that non-attachment is psychologically and socially adaptive. We used a Spanish validated version with adequate properties in meditators (α = 0.95) and non-meditators (α = 0.94) (Feliu-Soler et al., 2016).
- *Experiences Questionnaire* (EQ; Fresco, 2007): This 11-item questionnaire was designed to measure the capacity to observe one's thoughts and feelings as temporary and objective events of the mind, also known as “decentring.” Items from the scale are rated on a Likert scale ranging between 1 (never or very rarely true) and 5 (very often or always true), with higher scores indicating greater decentring. The Spanish validated version of the EQ has been shown to have good psychometric properties (α = 0.89) (Soler, 2014a).
- *Five Facets Mindfulness Questionnaire* (FFMQ; Baer, 2006): This measure consists of 39 items that assess five facets of mindfulness. Items are rated on a Likert scale ranging between 1 (never or very rarely true) and 5 (very often or always true), with higher scores meaning higher self-reported mindfulness skills. The Spanish version of the FFMQ has been validated with the facets of “observing” (α = 0.81), “describing” (α = 0.91), “acting aware” (α = 0.89), “non-judging” (α = 0.91), and “non-reactivity” (α = 0.80) (Cebolla, 2012).
- *Self-Other Four Immeasurables* (SOFI; Kraus and Sears, 2009): This is a 16-item rating scale designed to measure the application of the four immeasurable qualities at the heart of Buddhist teachings (loving kindness, compassion, joy, and acceptance toward both the self and others). We used a translated Spanish version of the original scale (Kraus and Sears, 2009), with adequate psychometric properties in all the dimensions: “positive-self” (α = 0.86), “negative-self” (α = 0.85), “positive-others” (α = 0.80), and “negative-others” (α = 0.82).
- *MINDSENS* (Soler, 2014b): This is a 19-item self-report index whose items are obtained from the FFMQ (Nos 1, 19, 20, 21, 24, 26, 29, 31, 33, 36) and EQ (Nos 2, 3, 4, 5, 7, 8, 9, 10, 11). This is not a different questionnaire; rather, is a composite of the two above-mentioned instruments that shows the strongest response to practice. The MINDSENS (α = 0.91) has been able to discriminate daily meditators from non-meditators in 82.3% of cases (Soler, 2014b).
- *Positive and Negative Affect Schedule* (PANAS; Watson et al., 1988): This instrument measures mood or emotion. It comprises 20 items, with 10 items for “positive affect” (e.g., inspired; α = 0.91) and 10 items for “negative affect” (e.g., afraid; α = 0.89). Each item is rated on a Likert scale, ranging between 1 (very slightly) and 5 (extremely), to measure the extent to which the affect has been experienced. We asked the participants to rate their affect during the previous month using a validated Spanish version (López-Gomez et al., 2015). An “affect balance” index of “positive affect—negative affect” was also used (Diener et al., 2012).
- *Satisfaction with Life Scale* (SWLS; Athay, 2012): This is a 5-item scale that measures general “satisfaction with life” using a Likert scale ranging between 1 (strongly disagree) and 7 (strongly agree). We used the Spanish validated version, which shows adequate psychometric properties (α = 0.88) (Vázquez et al., 2013).
- *Temperament and Character Inventory-Revised* (TCI-R-67; Cloninger, 1994): This is a 67-item self-report questionnaire, rated between 1 (completely disagree) and 5 (completely agree), that measures seven domains of personality: “novelty seeking,” “harm avoidance,” “reward dependence,” “persistence,” “self-directedness,” “cooperativeness,” “self-transcendence.” We used the corresponding Spanish validated version of this scale, with Cronbach's α-values ranging between 0.67 (reward dependence) and 0.86 (self-transcendence; Gutiérrez-Zotes, 2005).

### Procedure

After approval of the study by the corresponding University Ethical Board, individuals who had confirmed their presence at the retreat were sent a letter explaining the characteristics of the study and inviting them to participate in it. Some hours before the retreat opening ceremony, participants were assessed in a room at the monastery. At the end of the retreat, participants were assessed in the same place, or given the questionnaire to return within 48 h. The control group was selected from the list of participants in a master's degree course in mindfulness, and matched, as previously explained. The questionnaires were posted or presented during one of the course sessions both at baseline and 1 month later.

### Statistical analysis

Socio-demographic and practice variables of the sample were described, using means (SDs), or frequencies (percentages), depending on the nature of the variables. Comparisons between groups were performed using Student's *t*-tests and χ^2^-tests.

An analysis of covariance (ANCOVA) for repeated measures was performed, adjusting for baseline scores and hours of meditation practice (variable not matched, related to some of the study outcomes, in contrast to the rest of socio-demographics, which did not show any significant relationship with them). Cohen's *d* was used to estimate effect sizes, corrected for the mean dependence of the repeated-measures (Morris and DeShon, 2002). Contrasts between pre- and post-test measurements were performed using the corresponding *t*-test. The percentage of the pre-post increment (Δ%) was also calculated. Multiple linear regression models were estimated, using summed and change scores to test the possible mediating and/or moderating role of non-attachment. The mediating role concerns the processes that produce a treatment effect, and the moderating role concerns factors that affect the magnitude of that effect. Mediation is indicated if the difference in the dependent variable (Y_D_) depends on the difference in the independent variable (X_D_). Moderation is indicated if Y_D_ depends on the sum of the independent variable (X_S_). Therefore, both moderation and mediation can be assessed by using multiple regression models in which Y_D_ is regressed on two predictors at the same time: X_D_ (mediating effect) and X_S_ (moderating effect) (Judd et al., 2001). Standardized Beta coefficients were used to assess the individual contribution of the X_D_ and X_S_ predictors to explain Y_D_, and the Wald test was used to evaluate their statistical significance. Adjusted multiple determination coefficients (*R*^2^) were calculated to observe the explanatory power of the models (Martínez-González, 2006).

All statistical tests were bilateral with a significance level of α < 0.05. The SPSS-19 statistical software package was used.

## Results

All of participants were adults of European ethnicity (Table 1). The retreat group was middle-aged, gender-balanced, and highly educated, as was the control group owing to matching. Retreatants were less frequently in a stable relationship than controls [retreatants: 4 (21.1%); controls: 10 (52.6%); *p* = 0.044], but this variable did not show significant associations with the outcomes considered. Hours of meditation practice did not show significant differences between groups [retreatants: Md = 15,464.47 h (*SD* = 14,576.93); controls: Md = 8828.84 h (3405.77); *p* = 0.068], although it showed significant associations (only) with “non-reactivity” (*r* = 0.36; *p* = 0.028), so that it was controlled in the corresponding ANCOVA model. BMI was higher in the retreat group than in controls, although not significantly [retreatants: Md = 25.15 (*SD* = 1.46); controls: Md = 23.96 (*SD* = 2.16); *p* = 0.055], and there were no associations with the outcomes. All the participants in both groups had focused meditation as their main practice (>80% of the total time devoted).

**Table 1:** **Characteristics of study participants**.

	**Total sample (*n* = 38)**	**Retreatants (*n* = 19)**	**Controls (*n* = 19)**	***p***
Age^M^, Md (*SD*)	52.63 (9.88)	53.11 (10.38)	52.16 (9.62)	0.772
Sex^M^, Male	20 (52.6)	10 (52.6)	10 (52.6)	1.000
Ethnic group^M^, European	38 (100)	19 (100)	19 (100)	1.000
Marital status, Married/partner	14 (36.8)	4 (21.1)	10 (52.6)	0.044
Education^M^, University	24 (63.2)	11 (57.9)	13 (68.4)	0.501
Body Mass Index, Md (*SD*)	24.52 (1.88)	25.15 (1.46)	23.96 (2.16)	0.055
Hand dominance, Left	6 (15.8)	2 (10.5)	4 (21.1)	0.374
Practice^M^, Focused meditation^*^	38 (100)	19 (100)	19 (100)	1.000
Hours of practice, Md (*SD*)	12,146.66 (10,969.06)	15,464.47 (14,576.93)	8828.84 (3405.77)	0.068

Figures represent frequencies, percentages (in brackets) and the p-value associated with a χ^2^ contrast between the retreat group and control group, except for “age,” “body mass index,” “hours of practice,” where the figures represent means, standard deviations and the p-value associated with a t-contrast. M, matched variables.

* *>80% time*.

### Meditation retreat effects

No significant differences were found in any of the outcomes between groups at baseline (Tables 2, 3). Compared to controls, retreatants showed an increase in “non-attachment” (*F* = 6.08; df = 1; *p* = 0.019), “observing” (*F* = 5.18; df = 1; *p* = 0.029), “MINDSENS” (*F* = 14.95; df = 1; *p* < 0.001), “positive-affect” (*F* = 12.21; df = 1; *p* = 0.001), “balance-affect” (*F* = 6.06; df = 1; *p* = 0.019), and “cooperativeness” (*F* = 15.63; df = 1; *p* < 0.001). At the same time, compared to controls, retreatants showed a reduction in “describing” (*F* = 12.97; df = 1; *p* = 0.001), “negative-others” (*F* = 15.97; df = 1; *p* < 0.001), “reward dependence” (*F* = 13.43; df = 1; *p* = 0.001), and “self-directedness” (*F* = 11.97; df = 1; *p* = 0.001).

**Table 2:** **Meditation retreat effects on mindfulness variables**.

	**Retreatants**						**Controls**				
	**Pre**	**Post**	***p*^a^**	***d***	**Δ%**	**Pre**	**Post**	***p*^b^**	***d***	**Δ%**	***p*^c^**
Non-attachment^1^^,^^3^	122.58 (22.76)	136.26 (15.39)	0.001	−1.02	11.2	120.47 (17.48)	128.42 (11.73)	0.001	−1.11	6.6	0.019
Decentring^1^^,^^2^^,^^3^	39.37 (6.24)	42.32 (6.74)	0.019	−0.60	7.5	37.63 (5.55)	38.84 (4.95)	0.184	−0.32	3.2	0.114
Observing^1^^,^^2^^,^^3^	28.00 (5.81)	30.32 (4.90)	0.002	−0.88	8.3	26.58 (4.73)	27.58 (4.64)	0.028	−0.53	3.8	0.029
Describing^1^^,^^2^^,^^3^	27.74 (5.83)	25.47 (2.95)	0.048	0.59	−8.2	28.53 (5.55)	28.84 (5.00)	0.331	−0.24	1.1	0.001
Acting-aware^1^^,^^2^^,^^3^	28.00 (7.03)	29.58 (6.19)	0.131	−0.37	5.6	27.47 (5.86)	27.53 (5.44)	0.933	−0.02	0.2	0.128
Non-judging^1^^,^^2^^,^^3^	31.79 (6.45)	32.63 (3.38)	0.525	−0.17	2.6	30.68 (5.86)	30.11 (5.28)	0.265	0.27	−1.9	0.063
Non-reactivity^1^^,^^2^^,^^3^^*^	22.58 (4.11)	24.32 (4.44)	0.041	−0.51	7.7	21.00 (3.65)	22.05 (3.40)	0.328	−0.23	5.0	0.343
Positive-others^1^^,^^2^^,^^3^	13.95 (2.57)	14.26 (3.70)	0.789	−0.06	2.2	12.84 (1.97)	12.95 (2.59)	0.841	−0.05	0.9	0.238
Negative-others^1^^,^^3^	6.32 (2.79)	4.68 (1.05)	0.020	0.67	−26.0	6.53 (2.09)	7.00 (2.49)	0.252	−0.27	7.2	<0.001
Positive-self^1^^,^^2^^,^^3^	13.79 (2.97)	13.89 (2.36)	0.865	−0.04	0.7	12.42 (3.59)	13.47 (2.03)	0.334	−0.23	8.5	0.659
Negative-self^1^^,^^2^	6.89 (3.61)	6.26 (2.33)	0.281	0.20	−9.1	7.68 (2.64)	7.63 (2.94)	0.920	0.02	−0.7	0.163
MINDSENS^1^^,^^3^	50.00 (2.53)	52.89 (2.33)	<0.001	−1.88	5.8	48.95 (2.41)	49.26 (3.57)	0.591	−0.14	0.6	<0.001

Pre-, mean (SD) at pre-test. Post-, mean (SD) at post-test. d, Cohen's d. Δ%, percentage increment.

a pre-post t-contrast for retreat group.

b pre-post t-contrast for control group.

c ANCOVA for repeated measures using basal levels as covariate. Assumptions:

1 independent samples.

2 homoscedasticity.

3 normality.

* *controlling hours of practice*.

**Table 3:** **Meditation retreat effects on well-being and personality**.

	**Retreatants**					**Controls**					
	**Pre**	**Post**	***p*^a^**	***d***	**Δ%**	**Pre**	**Post**	***p*^b^**	***d***	**Δ%**	***p*^c^**
Positive-affect^1^,^3^	31.95 (5.83)	34.90 (6.35)	0.013	−0.57	9.2	30.79 (5.02)	30.32 (2.79)	0.428	0.12	−1.5	0.001
Negative-affect^1^,^3^	17.95 (6.63)	16.16 (6.65)	0.298	0.25	−10.0	17.79 (5.94)	17.63 (5.71)	0.660	0.11	−0.9	0.319
Balance-affect^1^,^3^	14.00 (10.01)	18.74 (11.22)	0.048	−0.45	33.9	13.00 (9.24)	12.68 (7.01)	0.678	0.07	−2.5	0.019
Life-satisfaction^1^,^2^,^3^	21.68 (7.63)	23.21 (4.60)	0.228	−0.34	7.1	21.05 (5.94)	21,95 (5.14)	0.293	−0.26	4.3	0.392
Novelty-seeking^1^,^2^,^3^	22.16 (3.59)	21.63 (6.97)	0.711	0.10	−2.4	20.74 (3.38)	20.68 (3.51)	0.921	0.03	−0.3	0.840
Harm-avoidance^1^,^2^,^3^	21.00 (6.24)	20.11 (6.25)	0.098	−0.51	−4.2	22.37 (5.62)	22.00 (5.15)	0.505	0.16	−1.7	0.360
Reward-dependence^1^,^2^,^3^	21.89 (6.70)	18.95 (6.25)	0.002	0.80	−13.4	23.00 (5.86)	23.26 (5.35)	0.635	−0.11	1.1	0.001
Persistence^1^,^2^,^3^	23.37 (4.15)	25.89 (4.38)	0.015	−0.62	10.8	24.84 (5.10)	25.74 (5.69)	0.255	−0.28	3.6	0.282
Self-directedness^1^,^3^	19.95 (6.15)	16.16 (4.61)	0.008	0.70	−19.0	20.95 (4.73)	20.32 (3.59)	0.293	0.28	−3.0	0.001
Cooperativeness^1^,^2^,^3^	30.37 (5.06)	32.26 (4.28)	0.004	−0.79	6.2	30.26 (4.40)	28.53 (3.35)	0.098	0.41	−5.7	<0.001
Self-trascendence^1^,^2^,^3^	28.53 (6.36)	29.26 (6.92)	0.374	−0.21	2.6	27.53 (6.05)	29.21 (5.76)	0.106	−0.40	6.1	0.540

Pre-, mean (SD) at pre-test. Post-, mean (SD) at post-test. d, Cohen's d. Δ%, percentage increment.

a pre-post t-contrast for retreat group.

b pre-post t-contrast for control group.

c ANCOVA for repeated measures using basal levels as covariate. Assumptions:

1 independent samples.

2 homoscedasticity.

3 *normality*.

### Mediating and/or moderating role of non-attachment

As observed in Table 4, “non-attachment” had a “mediating” role in “decentring” (Beta = 0.67; *R*^2^ = 0.34; *p* < 0.001), “acting-aware” (Beta = 0.40; *R*^2^ = 0.15; *p* = 0.034), “non-reactivity” (Beta = 0.51; *R*^2^ = 0.20; *p* = 0.006), “negative-affect” (Beta = −0.40; *R*^2^ = 0.12; *p* = 0.034), “balance-affect” (Beta = 0.41; *R*^2^ = 0.14; *p* = 0.032) and “self-directedness” (Beta = −0.40; *R*^2^ = 0.28; *p* = 0.020). Moreover, “non-attachment” had a moderating role in “describing” (Beta = −0.42; *R*^2^ = 0.13; *p* = 0.029), and “positive-others” (Beta = 0.55; *R*^2^ = 0.24; *p* = 0.003). Finally, it plays both a mediating and a moderating role in “satisfaction with life” [(mediating: Beta = 0.36; *p* = 0.023); (moderating: Beta = 0.36; *p* = 0.025); *R*^2^ = 0.39].

**Table 4:** **Moderating/mediating effects of non-attachment in mindfulness, well-being, and personality**.

**Variables**	**Effects**	**Beta**	***p***	***R*^2^**
Decentring	Moderating	0.26	0.115	0.34
	Mediating	0.67	<0.001	
Observing	Moderating	0.17	0.364	0.09
	Mediating	0.35	0.065	
Describing	Moderating	−0.42	0.029	0.13
	Mediating	−0.24	0.190	
Acting-aware	Moderating	0.04	0.839	0.15
	Mediating	0.40	0.034	
Non-judging	Moderating	−0.05	0.801	0.09
	Mediating	0.27	0.157	
Non-reactivity	Moderating	0.20	0.255	0.20
	Mediating	0.51	0.006	
Positive-others	Moderating	0.55	0.003	0.24
	Mediating	0.17	0.319	
Negative-others	Moderating	0.19	0.295	0.15
	Mediating	−0.25	0.181	
Positive-self	Moderating	−0.15	0.446	0.02
	Mediating	−0.05	0.813	
Negative-self	Moderating	0.01	0.959	0.01
	Mediating	−0.01	0.945	
MINDSENS	Moderating	0.08	0.692	0.01
	Mediating	0.10	0.626	
Positive-affect	Moderating	0.35	0.071	0.09
	Mediating	0.24	0.210	
Negative-affect	Moderating	−0.20	0.272	0.12
	Mediating	−0.40	0.034	
Balance-affect	Moderating	0.33	0.081	0.14
	Mediating	0.41	0.032	
Life-satisfaction	Moderating	0.36	0.025	0.39
	Mediating	0.36	0.023	
Novelty-seeking	Moderating	0.13	0.521	0.03
	Mediating	−0.06	0.768	
Harm-avoidance	Moderating	−0.07	0.741	0.01
	Mediating	−0.10	0.617	
Reward-dependence	Moderating	−0.28	0.149	0.08
	Mediating	−0.29	0.135	
Persistence	Moderating	0.06	0.766	0.04
	Mediating	0.21	0.274	
Self-directedness	Moderating	0.20	0.245	0.28
	Mediating	−0.40	0.020	
Cooperativeness	Moderating	0.17	0.379	0.05
	Mediating	0.24	0.215	
Self-trascendence	Moderating	−0.15	0.429	0.09
	Mediating	0.20	0.287	

*Moderating, moderating effect of non-attachment by using sum scores as a predictor of the difference in each dependent variable. Mediating, mediating effect of non-attachment by using difference scores as a predictor of the difference in each dependent variable. Beta, standardized slope of regression associated to each predictor. p, p-value related to Beta by using the Wald test. R^2^, determination coefficient (explained variance of the multivariate models)*.

## Discussion

To our knowledge, this is the first controlled study on the effect of a 1-month, intensive Vipassana meditation retreat on a wide array of psychological constructs related to mindfulness, psychological well-being and personality. We hypothesized that the retreat would increase mindfulness, well-being, and prosocial personality traits, and that psychological changes would be mediated and/or moderated by non-attachment. We have observed significant improvements in experienced meditators as a result of the retreat, showing a potential specific role for this type of training, which likely provides added benefits to daily and regular mindfulness practice. Moreover, a significant mediating and moderating role of non-attachment was observed, which is important from the perspective of the implied processes in improvements associated with meditative practice.

The socio-demographics reflected a pattern of highly educated, middle-aged participants of both genders, with many years of meditation experience. The only significant difference between the groups was that the retreatants were more frequently single than the controls. In any case, marital status showed no significant associations with the study outcomes, in contrast to meditative experience, which was related to non-reactivity, and was therefore controlled in the corresponding case. Both samples showed similar educational levels, somewhat relevant given that FFMQ seems to be influenced by them (Baer, 2008). With regard to practice, focused meditation predominated in both samples.

Surprisingly, there were no significant differences in decentring, which has shown to improve with simple mindful breathing (Feldman et al., 2010). Likewise, there were no differences in acting aware, non-judging, and non-reactivity. These results could be explained as some mindfulness variables tend to stabilize with long-term meditation experience (“ceiling effect”), and an intensive retreat would not modify previous high levels (Soler, 2014b). However, there were improvements in non-attachment, which suggests that ceiling effects on this variable are more difficult to reach. In the same way, there were improvements in MINDSENS and observing, as well as reductions in describing and negative-others. Changes in MINDSENS reinforce the notion that this index is sensitive to an intensive meditation retreat, despite previous meditative experience. The increase in observing and decrease in describing might be explained by the vow of silence taken during the retreat. To our knowledge, there are no studies relating silence with these factors, but experts on meditation have associated external and internal (mental) silence with a reduction in the need for labeling everyday experiences (Manocha, 2013). Finally, some studies have confirmed that mindfulness could decrease anger, hostility, and aggression toward others (Borders et al., 2010).

With regard to psychological well-being, some studies have found that mindfulness training improves positive affect (Nyklicek and Kuijpers, 2008; Orzech, 2009), and reduce negative affect (Glück and Maercker, 2011; Tanay et al., 2012). However, to our knowledge, there have been no studies on the effect of an intensive mindfulness retreat on these variables. In the present study, based on Vipassana techniques of meditation, there were no significant changes in satisfaction with life or negative affect. Nevertheless, retreatants showed an increase in their positive affect and affect balance.

We also found decreases in reward dependence and self-directedness, and increases in cooperativeness. Reward dependence refers to reinforcement and maintaining behaviors, and individuals scoring high in that variable tend to be sensitive and dependent on the approval of others (Cloninger et al., 1993). On the contrary, individuals with low scores in this trait are independent, practical and reserved (Cloninger et al., 1993; Maj, 2005). Cooperativeness refers to acceptance and identification with others, and includes compassion. The decrease in reward dependence could be associated with a reduction in the need for the approval of others, and this is compatible with an increase in cooperativeness, in the sense of being empathic, helpful and compassionate. Interestingly, the conditions of the retreat, in which verbal communication and social interactions were limited, increased proximity to others instead of independence and coldness. Previous studies have also found increases in compassion after mindfulness interventions (Birnie et al., 2010; Van Dam, 2011). However, the reduction in self-directedness seems somewhat unexpected, given its relation with purposefulness and discipline, characteristics needed in order to engage in a 1-month retreat. A possible explanation may be that high scores would be required at the beginning of the retreat, but not at the end. Moreover, the self-directedness scores after the retreat did not reflect being aimless or undisciplined (Svrakic, 2002; Nyklicek and Kuijpers, 2008), although it is true that this result contrasts with previous studies in which increases in self-directedness were found (Campanella, 2014; Crescentini and Capurso, 2015). These studies were conducted in non-retreat conditions, and subjects were therefore not free of life responsibilities, unlike during the retreat. In summary, these results are coherent with the reductions obtained in negative-others, and are also in line with other studies carried out in retreat conditions (Maj, 2005; Birnie et al., 2010).

Our findings showed that non-attachment played an important role in improving psychological outcomes such us mindfulness, well-being, and personality. Non-attachment has been proposed as a mechanism of mindfulness associated with long-term practice (Hölzel, 2011). We did not find this kind of association in our study, but we observed that the higher the basal levels for non-attachment, and the higher the improvement in this variable by means of meditative practice, the greater the benefits it might bring. We found that both retreatants and controls had similar non-attachment scores at baseline (similar to other samples of meditators) (Feliu-Soler et al., 2016), and the retreat was able to increase non-attachment by almost double in comparison with the control group, in terms of the increment. Therefore, this variable, to some extent, may have a ceiling effect when practiced in a non-intensive way. In short, non-attachment could be the keystone that mediates and moderates other changes produced by meditation, and these effects might wear off as the result of a ceiling effect in non-intensive practices. This is coherent with the teachings of experienced meditators that higher meditation states are only achieved by intensive meditation practices and retreats (Karmapa, 1981). Mindfulness seems to be the first stage of this process by means of which one is able to focus attention upon the flow of experience without distraction. However, through the intensive practice of specific meditation techniques, one will develop a greater ability to respond to this flow with equanimity, an impartial attitude in response to experience (Desbordes, 2015). Equanimity allows mindful awareness to be unbiased by facilitating an attitude of non-attachment, a perspective shift which includes certain sense of detachment from the ongoing experience (Gunaratana, 2002; Wallace, 2006). This perspective shift might be a cornerstone in the improvement of well-being by widening our perspective on experience, allowing us to respond skilfully to whatever is arising in the present moment (Desbordes, 2015).

The main limitation of this study was that it was not a randomized trial. This problem is very difficult to tackle in these types of studies because retreatants tend not to accept randomization to different conditions. The small sample size was also an important limitation, and could lead to a type II error. However, most of the existing research on retreats had been conducted with small groups owing to the high demands of the experience, and to the need for close supervision by the master leading the retreat. Another limitation was that the participants could show bias in their answers to the questionnaires to please the master or experimenters. Although the questionnaires were anonymous and the participants were supposed to be strongly committed to ethical values, this possibility cannot be completely excluded. On the other hand, the effects of the retreat may not be exclusively explained by meditation, as other possible active ingredients could be silence, a light vegetarian diet, group and master-disciple dynamics, general expectations, etc. The effect of these other ingredients are quite difficult to isolate in standard retreats if researchers intend to keep their ecological validity. The aim of the paper was the effect of a Vipassana meditation retreat, although we are fully aware that these other ingredients are always present in any retreat. Finally, this was a pre-post study without follow-up. Long-term follow-ups would be interesting because it is possible that the effects of mindfulness could vary over time, producing positive changes that are not immediately apparent after the retreat but which are evident after a longer period of time (Hill and Updegraff, 2012).

## Conclusions

A 1-month Vipassana meditation retreat may yield improvements in mindfulness, affect and personality, even in experienced meditators. Results suggest that non-attachment might facilitate these improvements. Further, studies should address some methodological flaws, such as randomization and sample size, in order to confirm our findings.

## Author contributions

JM, MD, and JG conceived and designed the study. MP, PH, and FR supervised the collection of data. JM analyzed and interpreted the data. JM, AC, JS, CV produced the drafting of the manuscript. JG and MD supervised all steps in the study. All authors provided a critical revision of the manuscript.

### Conflict of interest statement

The authors declare that the research was conducted in the absence of any commercial or financial relationships that could be construed as a potential conflict of interest.
