# Corrigendum: Psychological Effects of a 1-Month Meditation Retreat on Experienced Meditators: The Role of Non-attachment

**DOI:** 10.3389/fpsyg.2020.00852

**Published:** 2020-05-05

**Authors:** Jesús Montero-Marin, Marta Puebla-Guedea, Paola Herrera-Mercadal, Ausias Cebolla, Joaquim Soler, Marcelo Demarzo, Carmelo Vazquez, Fernando Rodríguez-Bornaetxea, Javier Garcia-Campayo

**Affiliations:** ^1^Faculty of Health and Sport Sciences, Primary Care Prevention and Health Promotion Research Network, Zaragoza, Centro de Investigación Biomédica en Red de Salud Mental, Zaragoza, Spain; ^2^Centro de Investigación Biomédica en Red de Salud Mental, Primary Care Prevention and Health Promotion Research Network, Instituto Aragonés de Ciencias de la Salud, Zaragoza, Spain; ^3^Department of Personality, Evaluation and Psychological Treatment, Universitat de València, Valencia, Spain; ^4^CIBERObn Ciber Physiopathology of Obesity and Nutrition, Santiago de Compostela, Spain; ^5^Servei de Psiquiatria, Hospital de la Santa Creu i Sant Pau (Barcelona), Departamento de Psicologia Clínica i de la Salut, Universitat Autònoma de Barcelona, Barcelona, Spain Centro de Investigación Biomédica en Red de Salud Mental, CIBERSAM, Madrid, Spain; ^6^Mente Aberta – Brazilian Center for Mindfulness and Health Promotion, Department of Preventive Medicine, Universidade Federal de Sao Paulo, Sao Paulo, Brazil; ^7^Hospital Israelita Albert Einstein, Sao Paulo, Brazil; ^8^Professor of Psychopathology, Universidad Complutense de Madrid, Red PROMOSAM, Valencia, Spain; ^9^Psychologist and Vipassana Master, President of Baraka Institute, San Sebastián, Spain; ^10^Miguel Servet Hospital and University of Zaragoza, Primary Care Prevention and Health Promotion Research Network, Instituto de Investigación Sanitaria Aragón (IIS Aragon), Centro de Investigación Biomédica en Red de Salud Mental, Zaragoza, Spain

**Keywords:** meditation, Vipassana, retreat, wellbeing, positive psychology, personality

In the published article, there was an error in affiliation 10. Instead of “Miguel Servet Hospital and University of Zaragoza, RedIAPP, Instituto Aragonés de Ciencias de la Salud, Zaragoza, Centro de Investigación Biomédica en Red de Salud Mental, Zaragoza, Spain,” it should be “Miguel Servet Hospital and University of Zaragoza, Primary Care Prevention and Health Promotion Research Network, Instituto de Investigación Sanitaria Aragón (IIS Aragon), Centro de Investigación Biomédica en Red de Salud Mental, Zaragoza, Spain.”

There was also an error in the Acknowledgments. The following sentence was removed as it was incorrect, “The project received funding from the Network for Prevention and Health Promotion in Primary Care (RD12/0005) grant from the Instituto de Salud Carlos III of the Spanish Ministry of Economy and Competitiveness and was cofinanced with European Union ERDF funds.” The correct funding from Instituto de Salud Carlos III has been added and the fully corrected Acknowledgments appears below.

## Acknowledgments

This study has been funded by Instituto de Salud Carlos III through the project RD12/0005/0006 (Co-funded by European Regional Development Fund “Una manera de hacer Europa”). The authors thank Vipassana Master Dhiravamsa for his permission and support to carry out the present study, which was partially funded by a MINECO grant PSI2012-35500. CIBEROBN is an initiative of the ISCIII.

The authors apologize for this error and state that this does not change the scientific conclusions of the article in any way. The original article has been updated.

